# Utility of a novel wearable electrode embedded in an undershirt for electrocardiogram monitoring and detection of arrhythmias

**DOI:** 10.1371/journal.pone.0273541

**Published:** 2022-08-23

**Authors:** Kazuaki Amami, Akiomi Yoshihisa, Yuko Horikoshi, Shinya Yamada, Takeshi Nehashi, Naoko Hijioka, Minoru Nodera, Takashi Kaneshiro, Tetsuro Yokokawa, Tomofumi Misaka, Yasuchika Takeishi

**Affiliations:** 1 Department of Cardiovascular Medicine, Fukushima Medical University, Fukushima, Japan; 2 Department of Clinical Laboratory Sciences, Fukushima Medical University School of Health Science, Fukushima, Japan; 3 Department of Arrhythmia and Cardiac Pacing, Fukushima Medical University, Fukushima, Japan; Centro Cardiologico Monzino IRCCS, ITALY

## Abstract

**Background:**

A 12-lead electrocardiogram (ECG) and Holter ECG have been established as gold standards for detection of arrhythmias. Recently, wearable ECG monitoring devices have been available. Our purpose of the present study was to investigate whether a novel wearable electrode embedded in an undershirt is useful for ECG monitoring and detection of arrhythmias.

**Methods:**

We studied 31 consecutive hospitalized patients who underwent catheter ablation of tachyarrhythmias. Patients equipped a wearable electrode and a lead CM5 of Holter ECG simultaneously, and total heart beats, maximum heart rate (HR), mean HR, minimum HR, detections of arrhythmias, such as atrial fibrillation, non-sustained ventricular tachycardia, and premature ventricular contractions (Lown’s grade >II), were compared between the two methods using a Holter ECG analysis software.

**Results:**

Median recording time of ECG by wearable electrodes was 12.6 hours. Strong correlations between the two methods were observed in total heart beats (R = 0.999, P <0.001), maximum HR (R = 0.997, P <0.001), mean HR (R = 0.999, P <0.001), minimum HR (R = 0.989, P <0.001) and QRS duration (R = 0.900, P <0.001). Bland-Altman analysis showed excellent concordance between each parameter measured by two methods. In addition, the detection of atrial fibrillation (nine events), non-sustained ventricular tachycardia (two events), and premature ventricular contractions of Lown’s grade >II (five events) were concordant in two methods. In addition, there were no significant difference in parameters of time-domain and frequency-domain analyses of heart rate variability between the two methods.

**Conclusions:**

The usefulness of a novel electrode embedded in an undershirt is equivalent to that of a Holter ECG in monitoring the ECG and detection of arrythmias.

## 1. Introduction

**Fig 1:**
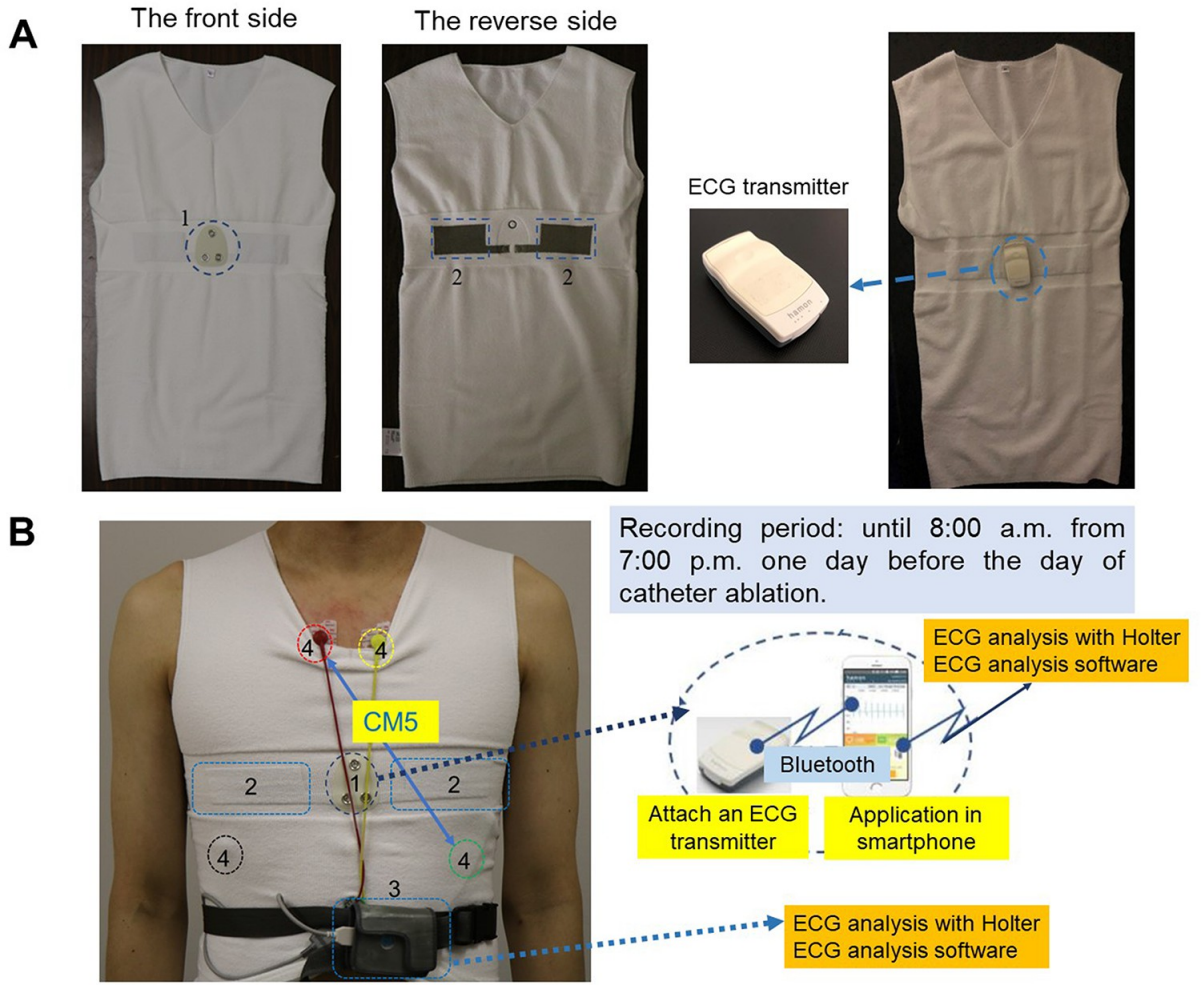
The structure and ECG acquiring systems of a wearable electrode embedded in an undershirt. (A) The structure and ECG acquiring systems of wearable electrodes. Left two panels show the front and reverse side of a wearable electrode. Right two panels show an ECG transmitter attached to an undershirt. (B) The method to record and send ECG using a wearable electrode and Holter ECG simultaneously. The explanation of components of a wearable electrode and Holter ECG: (1) The part which is connected to the ECG transmitter on an undershirt; (2) Silver textile electrode parts embedded in an undershirt; (3) Holter monitor; (4) ECG patch of Holter monitor. Abbreviations: ECG, electrocardiogram.

Detection of arrhythmias, such as atrial fibrillation and ventricular tachyarrhythmias, is important for early therapy and prevention of complications (e.g. stroke, heart failure, and sudden death) [1–3]. A 12-lead electrocardiogram (ECG) and Holter ECG are standard tools for detection of arrhythmias, however, these arrhythmias may not always be detected by these devices in hospitals or clinics, and some patients have episodes of arrhythmias without symptoms, such as asymptomatic atrial fibrillation, which lead to delay in ECG examinations. Therefore, alternative devices that can record ECG during more extended period are required for early diagnosis [4,5]. Recently, wearable ECG monitoring devices, such as Apple Watch and implantable cardiac monitors, are emerging for detection of arrhythmias [6–8]. These devices enable us to record ECG for a long-term period and increase the possibility of detecting arrhythmias. However, users of Apple Watch need to be aware of their arrhythmic events and operate the device during arrhythmias by themselves for recording ECG of the event, and recording time is limited within each 30 seconds. Although implantable cardiac monitors are effective for ECG monitoring during a long-term period to detect rarely occurring arrhythmias, their procedure is moderately invasive [9,10]. Hereby, it seems to be important for wearable ECG monitoring devices to monitor ECG accurately and continuously with less-invasive and easy methods. Recently, a wearable electrode embedded in an undershirt (Plum SENSE^®^, Mitsufuji Corp., Tokyo, Fig 1A) became available. A form as an undershirt, which is used daily in our lifestyle, may be appropriate for sustainable monitoring of ECG in daily life. However, abilities to measure heart rate (HR) and detect arrhythmias of wearable devices as undershirts have not been clearly verified. The purpose of the present study was to investigate whether a novel wearable electrode is useful for ECG monitoring and detection of arrhythmias compared to Holter ECG.

## 2. Materials and methods

### 2.1. Study population

The present study prospectively enrolled 31 patients who were hospitalized for catheter ablation of tachyarrhythmias at Fukushima Medical University Hospital between October 2020 and December 2020. The baseline characteristics of the patients are listed in Table 1. All patients were hospitalized one day before the catheter ablation, and equipped a wearable electrode and a Holter ECG simultaneously on the day of admission. Written informed consent was obtained from all study subjects, and the study protocol was approved by the Ethics Committee of Fukushima Medical University (approval number: 2020–122). Sample size was defined as of 30, based on statistical recommendations for pilot study [11,12].

**Table 1:** Baseline characteristics of the study subjects.

N	31
Age (years)	67.0 (57.0–73.0)
Male (n, %)	26 (83.9%)
Body mass index (kg/m^2^)	24.4 ± 4.6
Etiologies which catheter ablation was planned for	
Paroxysmal atrial fibrillation (n, %)	18 (58.1%)
Persistent atrial fibrillation (n, %)	7 (22.6%)
Atrial tachycardia (n, %)	1 (3.2%)
Paroxysmal supraventricular tachycardia (n, %)	3 (9.7%)
Premature ventricular contraction (n, %)	1 (3.2%)
Non-sustained ventricular tachycardia (n, %)	1 (3.2%)

### 2.2. Structure and systems of a wearable electrode embedded in an undershirt (Plum SENSE^®^)

A wearable single lead electrode embedded in an undershirt (Plum SENSE^®^, Mitsufuji Corp., Tokyo, Fig 1A) was used for the present study. This wearable device, which is made of polyester and polyurethane, can be used as daily clothing by exchanging and washing undershirts. On the middle chest of the reverse side of the undershirt, there are two silver textile electrode parts. These electrode parts are similar to lead I of a 12-lead ECG. An ECG transmitter (PS201-01, Mitsufuji Corp., Tokyo) is attached between the electrode parts for recording and sending ECG data.

### 2.3. ECG recording protocol

Patients equipped a wearable electrode and a Holter ECG (FM-960, Fukuda Denshi Co., Ltd., Tokyo) simultaneously for comparison of ability for ECG monitoring and detection of arrhythmias (Fig 1B). ECG was recorded until 8:00 a.m. from 7:00 p.m. one day before the catheter ablation. ECG patch of Holter ECG was attached to suprasternal notch and side chest avoiding contacts with electrode embedded in the undershirt. A lead CM5 of Holter ECG was chosen for reducing interference with electrode parts of the undershirt. The data recorded by a wearable electrode were sent from an ECG transmitter through Bluetooth communication to a smartphone via the application (Kawamata Kenko Ikiiki Apuri, Mitsufuji Corp., Tokyo). The ECG obtained from wearable electrodes were converted to a form for the analysis using a Holter ECG analysis software (SCM-850S, Fukuda Denshi Co., Ltd., Tokyo).

### 2.4. ECG analysis

Total heart beats, maximum heart rate (HR), mean HR, minimum HR, QRS duration, QTc interval corrected by Bazett’s formula, detections of arrhythmias, such as atrial fibrillation, non-sustained ventricular tachycardia, and premature ventricular contractions (Lown’s grade >II) were compared between wearable electrodes and the lead CM5 of Holter ECG, using the same Holter ECG analysis software by experienced clinical technologists who were blind to the clinical data. In addition, the time-domain analysis (average of NN-intervals, standard deviation of NN-intervals, standard deviation of 5-min averages of NN-intervals, average of the standard deviation of the 5-min NN intervals) and frequency-domain analysis including low-frequency power (LF; 0.046–0.140 Hz), high-frequency power (HF; 0.140–0.390 Hz) and LF to HF ratio of heart rate variability were compared between the two methods. For heart rate variability analyses, patients with atrial fibrillation and/or premature cardiac complex above 1000 per total recording time were excluded. The results of the ECG analysis were interpreted by independent cardiologists.

### 2.5. Statistical analysis

Normally distributed data were presented as mean ± standard deviation (SD), and were compared with a Student’s *t*-test. Non-normally distributed data were reported as median and interquartile range, and were compared with a Mann-Whitney *U* test. Differences in total heart beats, maximum HR, mean HR, minimum HR, QRS duration and QTc interval between wearable electrodes and Holter ECG were assessed with linear regression analysis and Bland-Altman analysis. The frequency-domain of heart rate variability was analyzed with two-way repeated measures analysis of variance, and the Huynh-Feldt ε correction was used to evaluate F ratios for repeated measures involving more than one degree of freedom. A value of P <0.05 was considered statistically significant. Statistical analyses were performed with the statistical software SPSS 28.0 (IBM Corp., Armonk, NY).

## 3. Results

### 3.1. Ability to measure HR, QRS duration, and QTc interval

**Fig 2:**
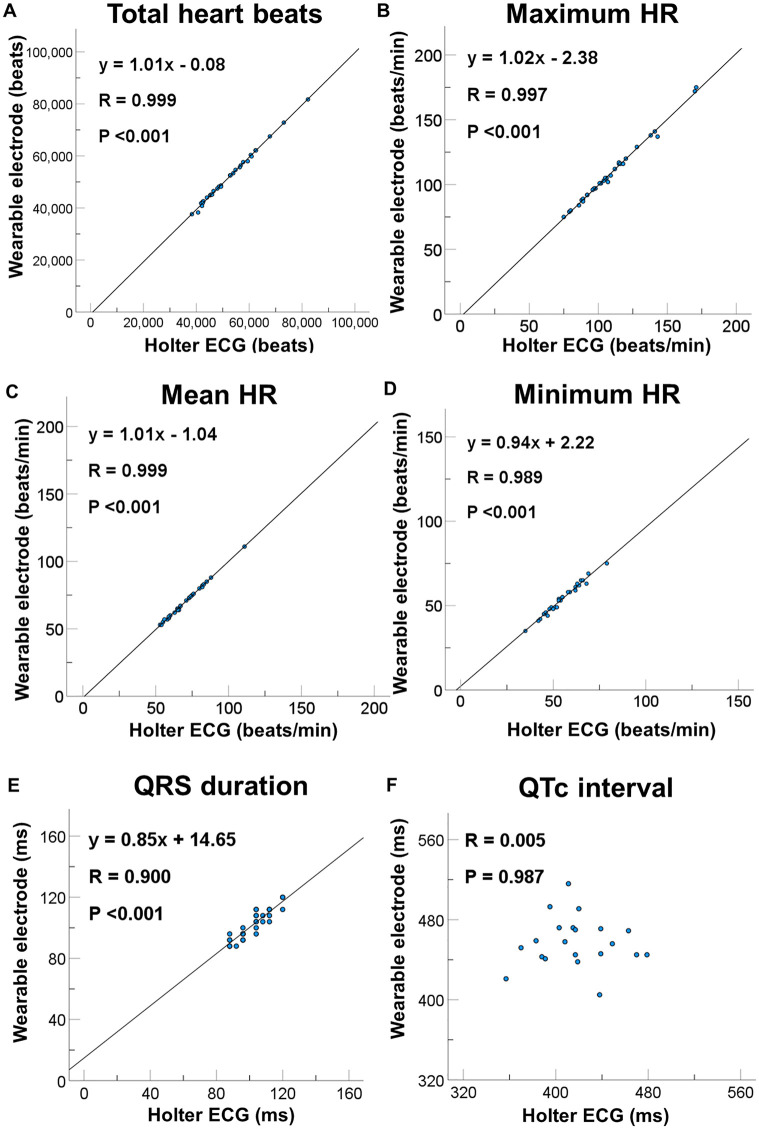
Regression analysis of each parameter measured by wearable electrodes embedded in undershirts and Holter ECG. (A) Regression analysis of total heart beats, (B) maximum HR, (C) mean HR, (D) minimum HR, (E) QRS duration and (F) QTc interval measured by two methods. Abbreviations: HR, heart rate; ECG, electrocardiogram.

**Fig 3:**
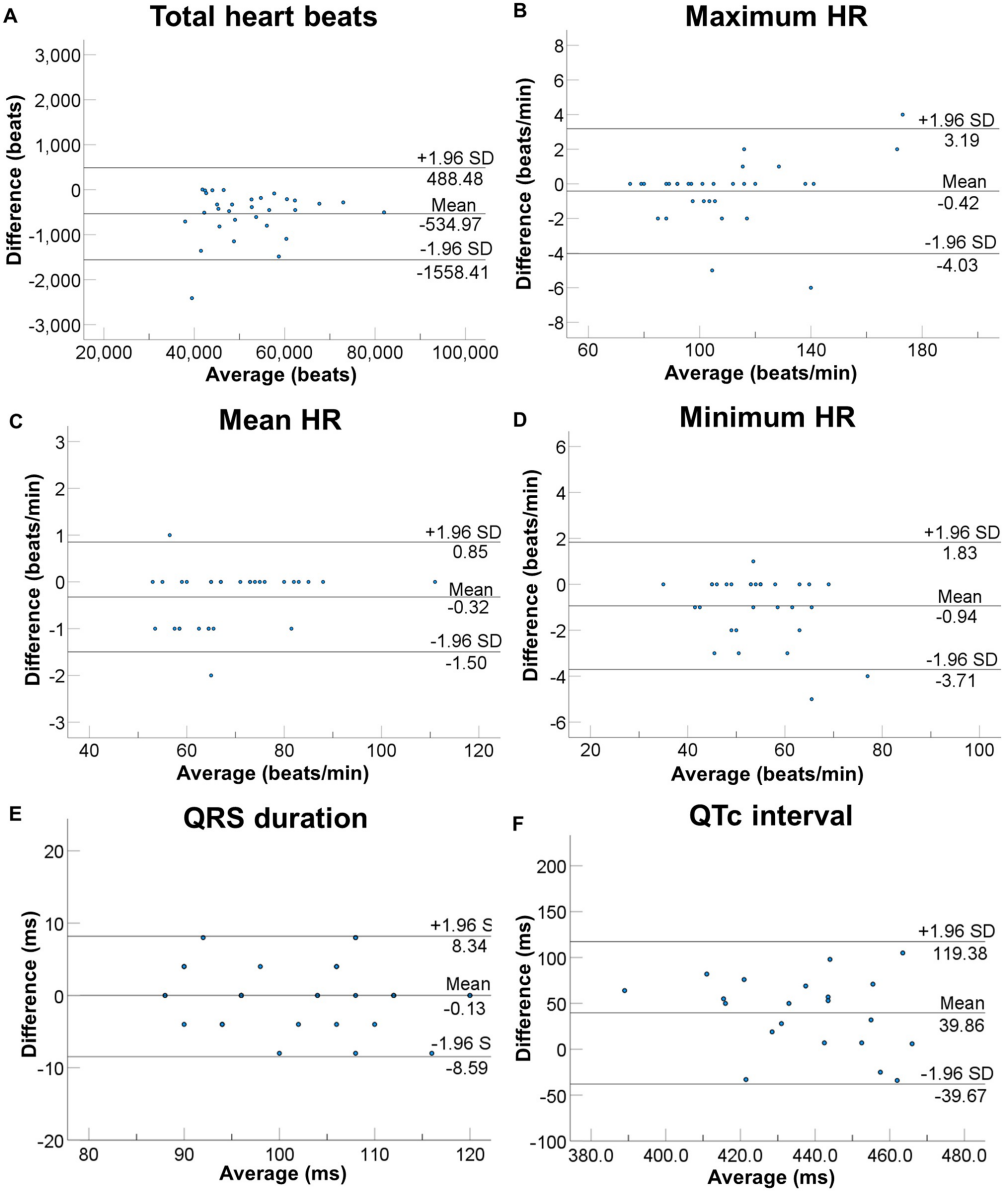
Bland-Altman plots of each parameter measured by wearable electrodes embedded in undershirts and Holter ECG. (A) Bland-Altman plots of total heart beats, (B) maximum HR, (C) mean HR, (D) minimum HR, (E) QRS duration and (F) QTc interval measured by two methods. Abbreviations: HR, heart rate.

We could acquire ECG from all of study subjects without recording failure. As shown in Table 2, total heart beats, maximum HR, mean HR, minimum HR and QRS duration were comparable between wearable electrodes embedded in undershirts and Holter ECG. Of 31 patients, QTc interval in 10 patients could not be measured because of flatness of QT interval. In 21 patients, QTc interval recorded by wearable electrodes were longer than those recorded by Holter ECG (457.5 vs. 417.7 ms, P<0.001). The linear regression analysis revealed strong correlations of total heart beats (y = 1.01x + 0.08, R = 0.999, P <0.001, Fig 2A), maximum HR (y = 1.02x + 2.38, R = 0.997, P <0.001, Fig 2B), mean HR (y = 1.01x + 1.04, R = 0.999, P <0.001, Fig 2C), minimum HR (y = 0.94x + 2.22, R = 0.989, P <0.001, Fig 2D), and QRS duration (y = 0.86x + 14.65, R = 0.900, Fig 2E) between two methods. However, QTc shows no significant correlation (R = 0.005, P = 0.987, Fig 2F). The Bland-Altman analysis showed mean differences were -534.97 ± 522.17 beats for total heart beats (Fig 3A), -0.42 ± 1.84 /min for maximum HR (Fig 3B), -0.32 ± 0.60 /min for mean HR (Fig 3C), -0.94 ± 1.41 /min for minimum HR (Fig 3D), -0.13 ± 4.32 ms for QRS duration (Fig 3E), and 39.86 ± 40.57 ms for QTc interval (Fig 3F). These results suggest that each parameter, except for QTc interval, measured by two devices presents excellent concordance.

**Table 2:** Median of total heart beats, maximum HR, mean HR, QRS duration, and average of minimum HR, QTc interval measured by wearable electrodes embedded in undershirts and Holter ECG.

N = 31	Wearable electrodes	Holter ECG	P value
Recording time (hours)	12.6 (12.1–13.2)	12.7 (12.1–13.2)	0.494
Total heart beats (beats)	48691.0 (44008.0–57980.0)	49364.0 (44020.0–59465.0)	0.699
Maximum HR (beats/min)	103.0 (92.0–117.0)	105.0 (92.0–118.0)	0.905
Mean HR (beats/min)	65.0 (59.0–76.0)	66.0 (59.0–76.0)	0.794
Minimum HR (beats/min)	53.9 ± 8.9	54.8 ± 9.4	0.689
QRS (ms)	96.0 (88.0–112.0)	104 (96.0–112.0)	0.261
QTc (ms)*	457.5 ± 24.8	417.7 ± 32.0	< 0.001

Abbreviations: HR, heart rate; ECG, electrocardiogram.

*QTc could be measured in 21 patients.

### 3.2. Detection of arrhythmias

**Fig 4:**
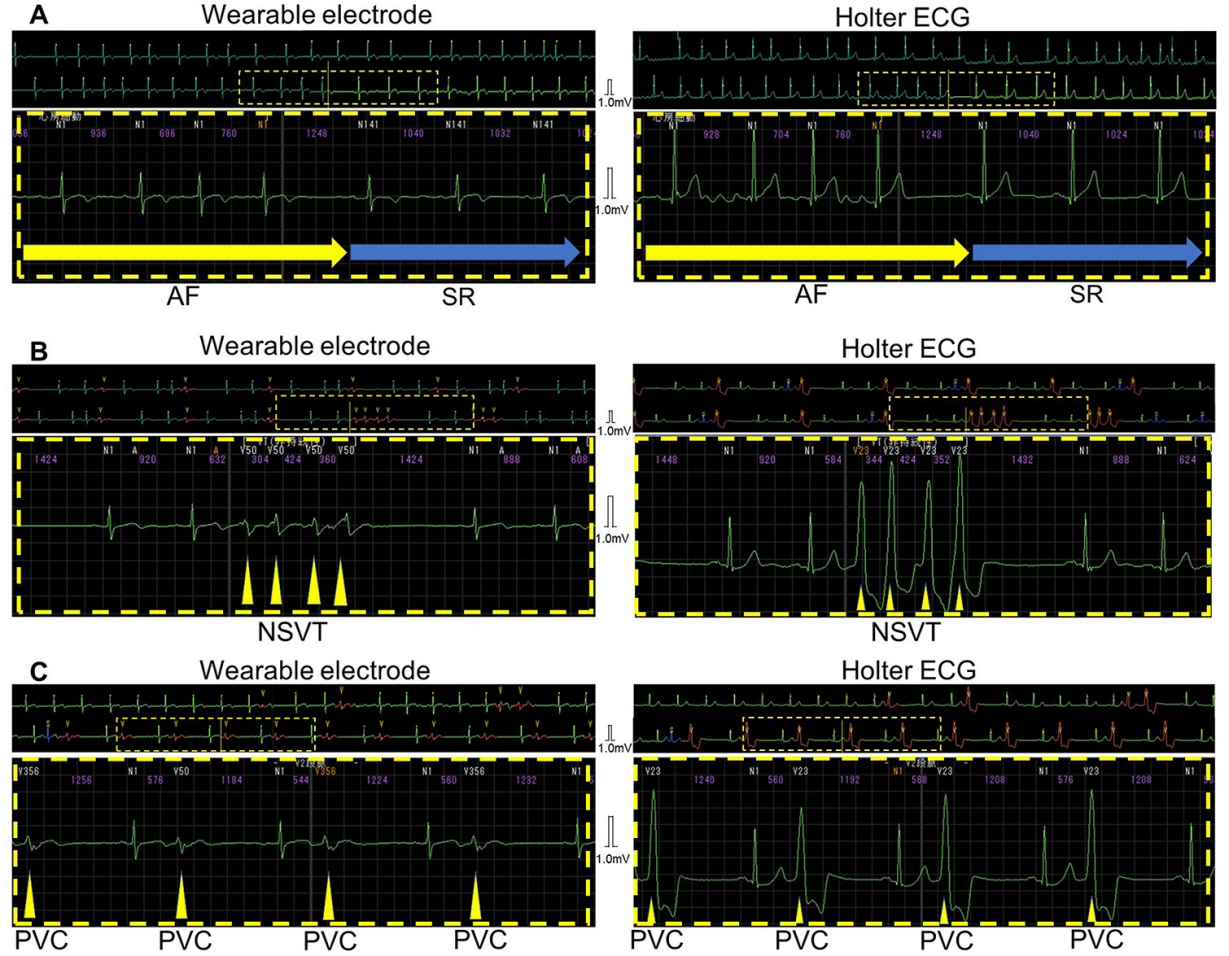
The comparison of wave patterns of arrhythmia between wearable electrodes embedded in undershirts and Holter ECG. (A) atrial fibrillation, (B) non-sustained ventricular tachycardia, and (C) premature ventricular contractions of Lown‘s grade >II. Abbreviations: AF, atrial fibrillation; SR, sinus rhythm; NSVT, non-sustained ventricular tachycardia; PVC, premature ventricular contractions; ECG, electrocardiogram.

Table 3 represents numbers of atrial fibrillation (nine events, Table 3A), non-sustained ventricular tachycardia (two events, Table 3B), and premature ventricular contractions of Lown’s grade >II (five events, Table 3C) which detected by wearable electrodes and Holter ECG. There were no discrepancies in the results of detection of arrythmias between two methods. The representative wave patterns of simultaneous recordings in same patients are illustrated in Fig 4 (A: atrial fibrillation, B: non-sustained ventricular tachycardia, C: premature ventricular contractions of Lown’s grade >II).

**Table 3:** Detection of arrhythmias. A. Detection of atrial fibrillation. B. Detection of non-sustained ventricular tachycardia. C. Detection of premature ventricular contractions of Lown’s grade >II.

**N = 31**		**Detection by wearable electrodes**	
		**Yes**	**No**
**Detection by** **Holter electrocardiogram**	**Yes**	9	0
	**No**	0	22
**N = 31**		**Detection by wearable electrodes**	
		**Yes**	**No**
**Detection by** **Holter electrocardiogram**	**Yes**	2	0
	**No**	0	29
**N = 31**		**Detection by wearable electrodes**	
		**Yes**	**No**
**Detection by** **Holter electrocardiogram**	**Yes**	5	0
	**No**	0	26

### 3.3. Comparisons of heart rate variability

**Fig 5:**
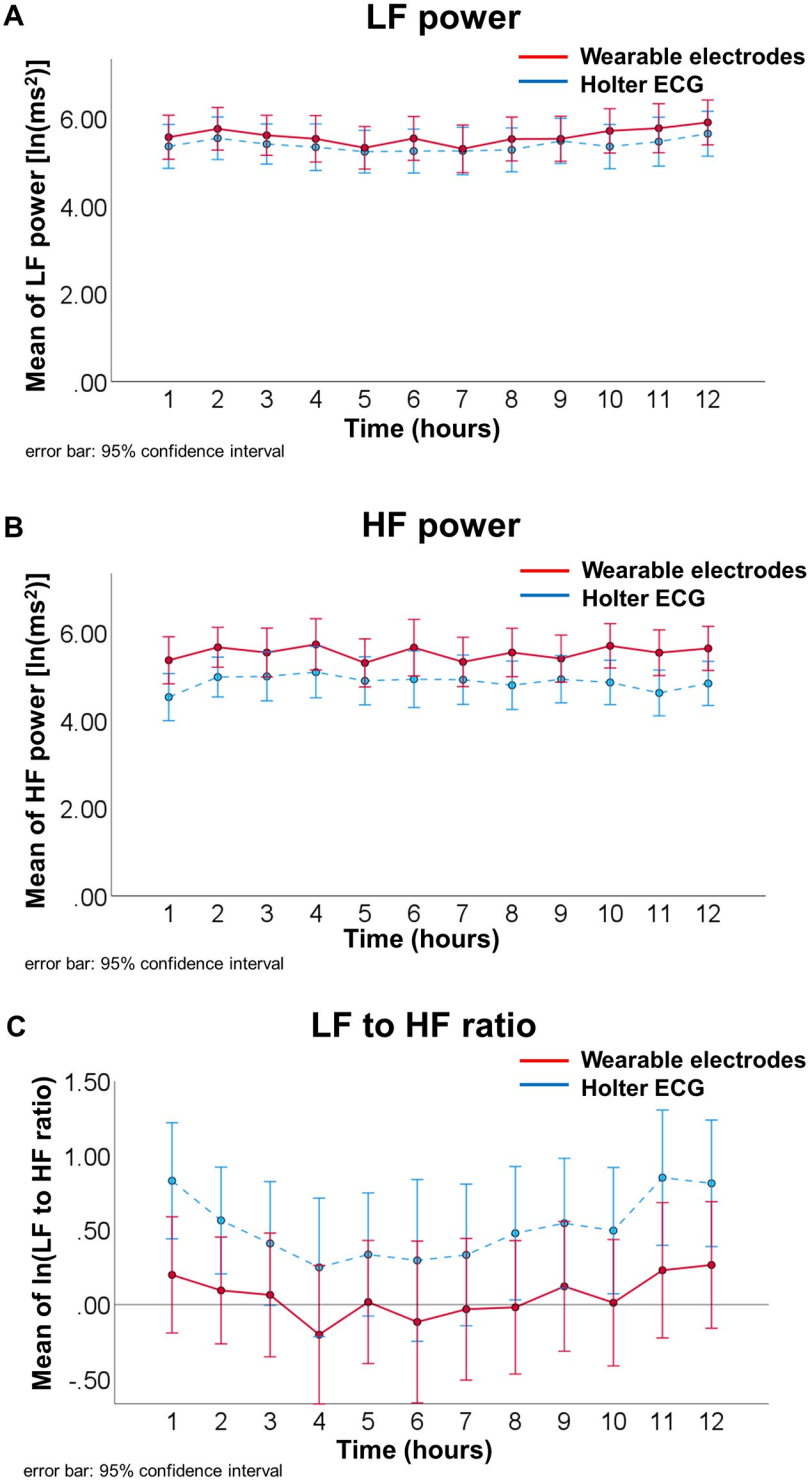
The frequency-domain analysis of heart rate variability analyzed every hour between wearable electrodes embedded in undershirts and Holter ECG. (A) LF power, (B) HF power and (C) LF to HF ratio. Abbreviations: LF, low-frequency; HF, high-frequency; ln, natural logarithm; ECG, electrocardiogram.

Of 31 patients, 17 patients with atrial fibrillation and/or premature cardiac complex above 1000 per total recording time were excluded. In 14 patients, the time-domain analysis during the hole recording time (Table 4) showed that average of NN-intervals, standard deviation of NN-intervals, standard deviation of 5-min averages of NN-intervals, average of the standard deviation of the 5-min NN intervals were comparable between the two methods. Fig 5 illustrates the frequency-domain analysis analyzed every hour, and two-way repeated measures analysis of variance was performed. There were no significant interactions between time course and difference of method in LF power (F_11, 286_ = 0.430, P = 0.869, ε = 0.586), HF power (F_11, 286_ = 0.827, P = 0.564, ε = 0.626) and LF to HF ratio (F_11, 286_ = 0.361, P = 0.913, ε = 0.586).

**Table 4:** Time-domain analysis of heart rate variability between wearable electrodes embedded in undershirts and Holter ECG.

N = 14	Wearable electrodes	Holter ECG	P value
AVNN (ms)	958.6 ± 114.4	958.1 ± 115.3	0.991
SDNN (ms)	95.6 (83.6–114.7)	95.9 (82.3–115.5)	1.000
SDANN (ms)	66.5 (47.3–75.7)	68.2 (49.8–75.9)	0.734
ASDNN (ms)	68.6 (61.0–85.4)	70.0 (57.1–84.5)	0.427

Abbreviations: ECG, electrocardiogram; AVNN, average of NN-intervals; SDNN, standard deviation of NN-intervals; SDANN, standard deviation of 5-min averages of NN-intervals; ASDNN, average of the standard deviation of the 5-min NN intervals.

## 4. Discussion

In the present study, we verified the abilities of a novel wearable electrode embedded in an undershirt: measuring ECG parameters, detection of arrhythmias and assessments of heart rate variability. As a result, this promising wearable device demonstrated similar ability compared to Holter ECG. These results imply clinical utility of the device.

### 4.1. Clinical utility of a wearable electrode embedded in an undershirt

Recently, early detection of arrhythmias is required for its therapy and prevention of complications [1,2,13]. There is a difficulty in detecting arrhythmias with several times of contacts with patients by a 12-lead ECG or Holter ECG. The developments in the field of ambulatory and remote ECG monitoring using wearable devices have come to be solutions for this problem [14,15]. It is important for these devices to be able to monitor ECG and detect arrhythmias with similar precision and accuracy compared to a gold standard device. In the present study, wearable electrodes embedded in undershirts showed comparable ability to Holter ECG for counting heart beats, measuring HR and QRS duration, and detection of arrhythmias using the same Holter ECG analysis software. There were strong correlations of total heart beats (R = 0.999), maximum HR (R = 0.997), mean HR (R = 0.999), minimum HR (R = 0.989) and QRS duration (R = 0.900) between the two methods, and Bland-Altman analysis showed excellent concordance between these parameters measured by two methods. On the other hands, wearable electrodes presented longer QTc interval compared to Holter ECG, and the result might be due to difference of sensing leads and heights of T wave. As shown in Fig 1, Wearable electrodes and lead CM5 of Holter ECG are resembles to lead I and V5 of 12-lead ECG respectively. QTc intervals were measured with tangent method automatically, and difference of T wave height could affect the result. With respect to the detection of arrhythmias, wearable electrodes showed a similar ability to Holter ECG for detection of atrial fibrillation, non-sustained ventricular tachyarrhythmia and premature ventricular contractions of Lown’s grade >II. In addition, the parameters of time-domain analyses and frequency-domain analyses measured by two methods were comparable.

It is also important for wearable devices to be easy to use without any expertise skills [16]. This novel wearable electrode is a kind of washable undershirt, and patients can continue to record and send ECG everyday by changing shirts, using the same ECG transmitter. Patients do not have to recognize arrhythmic events and perform any operation by oneself except for only wearing the undershirt to record the wave pattern. Therefore, wearable electrodes may be preferable for long-term ECG monitoring, and increase the opportunities of detecting arrhythmias. However, this wearable device has only single lead to record ECG, and we selected only one lead of Holter ECG to compare with. There are some studies, comparing a single lead ECG device with Holter ECG and these devices could be acceptable alternatives for ambulatory ECG monitoring in patients with arrhythmia [17–19]. In addition, the consideration to invasiveness of methods to monitor ECG is indispensable. Implantable cardiac monitors are effective for ECG monitoring during a long-term period to detect rarely occurring arrhythmias and recurrence of arrhythmias after catheter ablations [20–22], however, wearable electrodes may enable less-invasive ECG monitoring.

We could verify the utility of a novel wearable electrode embedded in an undershirt for ECG monitoring and detection of arrhythmias, and this wearable device may be preferable for long-term use because of its user-friendly characteristics. Further studies may be required to evaluate the ability of the device to detect arrhythmias during a long-term period.

### 4.2. Limitations

First, the current study was performed in a single center, and the numbers of study population and events were relatively small. Second, wearable electrodes only have single lead and we selected only lead CM5 of Holter ECG to compare with, therefore, we could not fully assess the differences in abilities to record ECG between wearable electrodes and Holter ECG with all leads. Wearable electrodes and lead CM5 of Holter ECG are resembles to lead I and V5 of 12-lead ECG respectively. The difference in lead may led to lower T wave of wearable electrodes and longer QTc interval [23] in wearable electrodes. Third, as the recording period of ECG was nighttime in hospital, there would have been a little body movement of the patients, and this possibly leads to underestimating the effect of motion artifacts. We are considering performing an additional study on these issues in future.

## 5. Conclusions

A novel wearable electrode embedded in an undershirt demonstrated abilities comparable to those of Holter ECG for ECG monitoring and detection of arrhythmias. This wearable device possibly provides clinical utilities for long-term ECG monitoring and detection of arrhythmias.

## Supporting information

S1 Data (XLSX)
